# Physician and patient-related factors associated with inappropriate prescribing to older patients within primary care: a cross-sectional study in Brazil

**DOI:** 10.1590/1516-3180.2020.0411.r1.18112020

**Published:** 2021-04-05

**Authors:** Welma Wildes Amorim, Luiz Carlos Passos, Romana Santos Gama, Renato Morais Souza, Lucas Teixeira Graia, Jéssica Caline Macedo, Djanilson Barbosa Santos, Marcio Galvão Oliveira

**Affiliations:** I MD, PhD. Internal Medicine Professor, Medicine Course, Department of Natural Sciences, Vitória da Conquista Campus, Universidade Estadual do Sudoeste da Bahia (UESB), Vitória da Conquista (BA), Brazil.; II MD, PhD. Internal Medicine Professor, Postgraduate Program on Medicine and Health, Department of Internal Medicine, Universidade Federal da Bahia (UFBA), Salvador (BA), Brazil.; III BPharm. Master's Student, Postgraduate Program on Medicine and Health, Universidade Federal da Bahia (UFBA), Salvador (BA), Brazil.; IV BPharm. Research Assistant, Multidisciplinary Health Institute, Anísio Teixeira Campus, Universidade Federal da Bahia (UFBA), Vitória da Conquista (BA), Brazil.; V BPharm. Research Assistant, Multidisciplinary Health Institute, Anísio Teixeira Campus, Universidade Federal da Bahia (UFBA), Vitória da Conquista (BA), Brazil.; VI BPharm. Master's Student, Master's Program on Collective Health, Multidisciplinary Health Institute, Anísio Teixeira Campus, Universidade Federal da Bahia (UFBA), Vitória da Conquista (BA), Brazil.; VII PhD. Epidemiology Professor, Department of Collective Health, Universidade Federal do Recôncavo da Bahia (UFRB), Santo Antônio de Jesus (BA), Brazil.; VIII BPharm, PhD. Evidence-Based Healthcare Professor, Master's Program on Collective Health, Multidisciplinary Health Institute, Anísio Teixeira Campus, Universidade Federal da Bahia, Vitória da Conquista (BA), Brazil.

**Keywords:** Aged, Polypharmacy, Brazil, Prescription drugs, Primary health care, Older adults, Potentially inappropriate medications, Primary care

## Abstract

**BACKGROUND:**

Physician and patient-related characteristics can influence prescription of medications to older patients within primary healthcare. Use of Brazilian criteria may indicate the real prevalence of prescription of potentially inappropriate medications to this population.

**OBJECTIVES:**

To evaluate prescription of potentially inappropriate medications to older patients within primary care and identify patient-related and prescribing physician-related factors.

**DESIGN AND SETTING:**

This cross-sectional study was conducted in 22 public primary care facilities in Brazil, among older people (≥ 60 years) who were waiting for medical consultations.

**METHODS:**

Interviews were conducted before and after the medical consultations. If the patient received a medical prescription at the consultation, all the drugs prescribed and the physician's medical council registration number were recorded. Prevalence ratios were estimated to ascertain the magnitude of prescription of potentially inappropriate medications, along with patient and physician-related factors associated with such prescription.

**RESULTS:**

In total, 417 older patients were included; 45.3% had received ≥ 1 potentially inappropriate medication, and 86.8% out of 53 physicians involved had prescribed ≥ 1 potentially inappropriate medication. The strongest patient-related factor associated with higher prevalence of prescription of potentially inappropriate medications was polypharmacy. Among physician-related factors, the number of patients attended, number of prescriptions and length of medical practice < 10 years were positively associated with prescription of potentially inappropriate medications.

**CONCLUSIONS:**

High prevalence of prescription of potentially inappropriate medications was observed. Physician-related characteristics can influence prescription of medications to older people within primary healthcare. This suggests that there is a need for interventions among all physicians, especially younger physicians.

## INTRODUCTION

Prescription of potentially inappropriate medications (PIMs) to older adults refers to the prescribing of medications that can potentially increase the risk of adverse health outcomes, given that safer and more effective treatment options are available for a particular indication. Prescription of PIMs may even refer to a situation in which the risks of therapy outweigh the benefits.^1^^–^^4^ Thus, prescribing practices have the biggest impact on occurrences of preventable adverse reactions due to the drug.^3^^,^^5^^–^^9^ Such occurrences impose significant burdens, like functional decline, falls, delirium and other geriatric syndromes,^5^^,^^10^^,^^11^ and lead to greater numbers of emergency department visits and hospital admissions, and higher mortality rate.^9^^,^^12^^,^^13^

In addition, the high cost of prescribing PIMs to older adults places a great economic burden on healthcare systems.^3^^,^^5^^,^^8^^,^^14^^–^^16^ Therefore, PIM prescription has become an important worldwide public health issue.^2^^,^^3^^,^^17^ In 2013, 37% of non-hospitalized older people in Canada received ≥ 1 prescription of PIM, equivalent to an estimated cost of US$ 75 per older person or a total cost of US$ 419 million in that year.^15^ Similarly, after starting to use at least one PIM, a quarter of older adults continued to use them beyond one year,^18^ thereby potentially increasing the risk of adverse events and raising healthcare costs.

Both, clinical and nonclinical factors may influence the prescription of medications. However, it is necessary to recognize factors that may influence clinical decisions, such as physician and patient-related characteristics. These can include the physician's length of medical practice and time in the specialty; and the patient's age, sex, income and comorbidities.^19^ In a systematic review of European studies, polypharmacy, advanced age and female sex were the patient-related factors that were most frequently associated with PIM prescription. Other factors that were less frequently associated with PIM prescription included depression, moderate self-rated health quality and poor functional and economic status.^17^

In Brazil, a previous study among community-dwelling older adults was conducted to assess the use of PIMs. It was noted that 34.5% of those older people used ≥ 1 PIM and that certain patient factors were associated with the use of inappropriate medication. These factors included drug use by illiterate older adults, black race, daily use of ≥ 4 medications, use of prescription drugs and acquisition of the drug through the Brazilian public healthcare system as opposed to acquisition in drug stores.^20^ However, only a few studies have hitherto evaluated associations of physician-related factors with PIM prescription.^19^^,^^21^^–^^23^ In some of those studies, it was noted that less time spent with patients and certain medical specialties were associated with higher likelihood of PIM prescription.^19^^,^^21^

Therefore, it is essential to understand the drivers of PIM prescription in order to be able to target specific interventions that might improve prescribing practices for the older adult population.

## OBJECTIVE

The aims of this study were to evaluate the frequency of prescription of potentially inappropriate medications to older patients within primary care, in accordance with Brazilian criteria, and to identify the factors associated with such prescriptions, among patients and their prescribing physicians.

## METHODS

### Study design

This cross-sectional study constituted the baseline component of a randomized clinical trial named “Development and evaluation of a mobile application for supporting prescription of appropriate medications to the elderly.”

### Setting

This study was conducted in all the public primary care facilities in the urban area of Vitória da Conquista (BA), Brazil, which included 15 family health units (FHUs) and 7 primary healthcare units (PHUs). These units belong to the Brazilian public healthcare system (Sistema Único de Saúde, SUS), which provides public healthcare at primary, secondary and tertiary levels to the entire population. Despite the availability of a complementary healthcare system that provides health insurance and private health care for individuals who can afford it, most Brazilian people are assisted only through SUS. The Family Health Strategy is a major program that delivers public primary healthcare to families in areas covered by FHUs. Each FHU has at least one multidisciplinary team that comprises general practitioner physicians, nurses, dentists, nursing technicians and community health workers. The PHUs in the city of Vitória da Conquista provides primary healthcare without the coverage of the Family Health Strategy.

### Data collection

Data were collected from all the public primary care facilities in the urban area of the city between September 2016 and December 2017. This was done using a multidimensional questionnaire adapted from the instrument used in the Health, Wellbeing and Aging in Latin America and the Caribbean (SABE) project.^24^ This questionnaire was then created in a digital data collection platform (KoBoToolbox, Cambridge, MA, United States). Participant interviews were conducted both before and after their medical consultations at the 22 primary care facilities mentioned above, and all these medical consultations were timed. After the consultations, if the patient had received a medical prescription, we recorded all drugs that had been prescribed and the physician's registration number in the regional medical council's registry. If the patient had any health-related interview impediments such as deafness or moderate-to-severe cognitive deficit, all the information was obtained from the person accompanying the patient.

### Study population

The patients eligible for inclusion were older people aged ≥ 60 years who were waiting for medical consultations in the facilities. They were invited to participate in the study and were included only after agreeing to this and signing an informed consent form. We excluded individuals who did not receive a medical prescription and those who had hearing impairments and/or severe cognitive deficits and were unaccompanied by a person with whom the interview could be conducted on the patient's behalf.

### Sample description

This study used a non-probabilistic sampling method and the participants were selected through consecutive sampling. The sample size was estimated at 513 participants, considering a confidence level of 95%, an acceptable difference of 0.05, an assumed proportion of PIM prescription (the event of interest) of 50% and an expected loss rate of 25%.

### Variables

The main outcome from the study (dependent variable) was a PIM prescription. The independent variables relating to patients included the following: 1) sociodemographic characteristics (i.e. sex, age, marital status, schooling, income, ethnicity and health insurance); 2) clinical characteristics (i.e. cognitive deficit, sensory deficits [auditory/visual], chronic pain, insomnia, multimorbidity, fall history, functional status and hospitalization); and 3) characteristics relating to medical care (i.e. having a companion attending consultations, consultation length ≤ 10 minutes and prescription of polypharmacy). The independent variables relating to physicians comprised sex, length of time in medical practice, specialty, type of primary care unit, number of patients attended per physician, number of prescriptions per physician and number of PIM prescriptions per physician.

### Measurement tools

The prescribed medications were analyzed in terms of the composition of their active ingredients. Prescriptions were considered to be PIMs based on the Brazilian consensus on potentially inappropriate medications for elderly people,^4^ with regard to rationale, clinical condition and exceptions.

The patient-related variables included the following:

1. Personal monthly income (the Brazilian minimum monthly wage corresponded to US$ 283.00 at the time of our current study).
2. Cognitive impairment, which was assessed using the Mini-Mental State Examination,^25^^,^^26^ considering different cutoff points according to educational level.^27^
3. Functional status, using the Katz Index of Activities of Daily Living^28^^,^^29^ and Pfeffer's Functional Assessment Questionnaire^30^^,^^31^ (the latter was applied only to the person accompanying the patient). These instruments were used to assess functional status in the form of the ability to perform activities of daily living (ADLs) and instrumental activities of daily living (IADLs), respectively.
4. Self-perceived visual impairment and auditory impairment.
5. History of falls: falls after reaching the age of 60 years.
6. Chronic pain, i.e. pain that lasted longer than 12 weeks.
7. Multimorbidity, defined as the presence of two or more self-reported chronic diseases.
8. Attending the consultation together with a companion, i.e. the presence of a person who accompanied the patient to the consultation.
9. Hospitalization, defined as hospital admission within the past 12 months.
10. Insomnia, i.e. difficulty in falling asleep or staying asleep.
11. Polypharmacy, defined as prescription of ≥ 5 medications.^32^

To determine the physician-related variables, the prescribing physicians were firstly identified through their registration numbers in the regional medical council's registry, which was available from the prescription forms that had been issued to the patients. From these registration numbers, we searched for the following data on the website of the medical council: sex, specialty and year in which the physician issued his or her first prescription. The latter was used to calculate the length of time for which the physician had been in medical practice, in years. The primary care units were classified as FHU or PHU.

### Statistical analysis

Descriptive analyses were performed on the variables. Associations between categorical variables were assessed using the chi-square test, and the prevalence ratio (PR) was measured to estimate the strength of the association. Means of the continuous variables were compared by means of the t test if the statistical assumptions were satisfied, or the Mann-Whitney U test if otherwise.

Multivariable analysis (Poisson regression) was used to adjust for potential confounders. Statistical significance was determined in terms of a 95% confidence interval (CI) or a P-value < 0.05. Data were considered to be missing from the analyses in the following cases: 1) the interviewer marked the option “unknown” in the questionnaire; 2) the physician did not have a registration number in the regional medical council's registry (e.g. if the physician was working in the “More Doctors” program); or 3) certain words and numbers were illegible (e.g. drug name in the prescription and regional medical council registration number). The R statistical software package was used to calculate the prevalence ratio, while all other analyses were undertaken using the SPSS software, version 25 (IBM Corp., Armonk, NY, United States).

### Ethics approval

This study was approved by the Research Ethics Committee of the Multidisciplinary Health Institute, Federal University of Bahia (technical opinion number 378.198; on August 30, 2013). All participants signed an informed consent statement at the time of their participation in the study.

## RESULTS

Out of the 522 eligible older people who were waiting for medical consultations in the primary care units, we included 417 cases who received medical prescriptions at their consultations. Among these 417 prescriptions, 189 had at least one PIM. The older people in this study had a median age of 69 years (**Table 1**); 67.4% of them were women; and 62.4% had a monthly income < 1 minimum wage. Regarding medical conditions, 50.2% had cognitive impairment, 67.9% suffered from chronic pain and 62.8% reported multimorbidity. Older people who received polypharmacy prescriptions and prescriptions with ≥ 1 PIM accounted for 16.8% and 45.3%, respectively.

**Table 1:** Sociodemographic and clinical characteristics of the study participants

Characteristics		Percentage (n/N)
**Sex**		
	Female	67.4 (281/417)
	Male	32.6 (136/417)
**Age in years, median (IQR)**		69.0 (64.0-75.0)
**Schooling**		
	Illiterate	43.1 (179/415)^*^
	Literate	56.9 (236/415)^*^
**Marital status**		
	Single, widowed or divorced	52.0 (217/417)
	Married	48.0 (200/417)
**Ethnicity**		
	White	24.9 (102/409)^*^
	Mixed	75.1 (307/409)^*^
**Personal income**		
	< 1 minimum monthly wage	62.4 (260/417)
	1 minimum monthly wage	37.6 (157/417)
**Health plan**		
	Yes	3.4 (14/417)
	No	96.6 (403/417)
**Cognitive impairment**		
	Yes	50.2 (209/416)^*^
	No	49.6 (207/416)^*^
**ADL impairment**		
	Yes	35.5 (148/417)
	No	64.5 (269/417)
**IADL impairment**		
	Yes	60.2 (50/83)^*^
	No	39.8 (33/83)^*^
**Visual impairment**		
	Yes	58.5 (245/417)
	No	41.2 (172/417)
**Hearing impairment**		
	Yes	34.5 (144/417)
	No	65.5 (273/417)
**Insomnia**		
	Yes	48.4 (201/415)^*^
	No	51.6 (214/415)^*^
**Chronic pain**		
	Yes	67.9 (83/4117)
	No	32.1 (134/417)
**Fall history**		
	No	41.3 (169/409)^*^
	Yes	58.7 (240/409)^*^
**Hospitalization**		
	Yes	12.2 (51/417)
	No	87.8 (366/417)
**Companion attending consultation**		
	Yes	20.0 (83/416)^*^
	No	80.0 (333/416)^*^
**Multimorbidity**		
	Yes	62.8 (262/417)
	No	37.2 (155/417)
**Polypharmacy**		
	Yes	16.8 (70/417)
	No	83.2 (347/417)
**PIM prescription**		
	Yes	45.3 (189/417)
	No	54.7 (228/417)
**Length of consultation ≤ 10 minutes**		
	Yes	52.8 (220/417)
	No	47.2 (197/417)

* Data missing. One minimum monthly wage was equivalent to US$ 283.00.

ADL = activity of daily living; IADL = instrumental activity of daily living; PIM = potentially inappropriate medication; IQR = interquartile range.

Prescriptions provided by 53 physicians were analyzed in this study (**Table 2**). Older men accounted for 51% of the physicians; those without a specialty constituted 75.5%; and the median length of medical practice was eight years. A total of 86.8% of the physicians prescribed at least one PIM, with a median of four PIMs prescriptions per physician.

**Table 2:** General characteristics of the prescribing physicians

Characteristics		Percentage (n/N)
**Sex**		
	Female	49.0 (25/51)*
	Male	51.0 (26/51)*
**Length of time in practice in years, median (IQR)**		9.0 (5.0-26.5)
**Specialty**		
	Yes	24.5 (12/49)**
	No	75.5 (37/49)**
**Type of primary care unit**		
	FHU	64.2 (34/53)
	PHU	35.8 (19/53)
**Number of patients attended per physician, median (IQR)**		11.0 (6.0-14.5)
**Number of prescriptions per physician, median (IQR)**		10.0 (5.0-12.0)
**PIM prescriptions**		
	Yes	86.8 (46/53)
	No	13.2 (7/53)
**Number of PIM prescriptions per physician, median (IQR)**		4.0 (2.0-5.0)
**Length of time in practice < 10 years**		
	Yes	47.2 (25/53)
	No	52.8 (28/53)

* Data missing due to illegibility of the physician's registration number in the medical council's registry, thus making it impossible to identify the physician's sex

** Data missing due to unavailability of the physician's registration number in the medical council's registry (for example, because of working in the “More Doctors” program).

IQR = interquartile range; PHU = primary healthcare unit; FHU = family health unit; PIM = potentially inappropriate medication.

In this study, a total of 1,281 drugs were analyzed, within 417 prescriptions. Forty-two per cent (538/1,281) of the medications prescribed were considered to be PIM-suspect medications. After analysis on the rationale, clinical condition and exceptions, 44.6% (240/538) of them were confirmed as PIMs (**Table 3**). The five most frequently prescribed pharmacological classes of PIMs were anti-inflammatory agents (22.5%), oral hypoglycemic agents (12.1%), systemic corticosteroids (10.0%), proton pump inhibitors (9.6%) and benzodiazepines (8.3%).

**Table 3:** Classes of potentially inappropriate medications most frequently prescribed to older people

Ranking	Pharmacological class	Specification	Percentage of potentially inappropriate prescriptions % (n/N)
1	Non-steroidal anti-inflammatory agent	Ibuprofen, meloxicam, ketoprofen	22.5 (54/240)
2	Hypoglycemic	Glyburide	12.1 (29/240)
3	Systemic corticosteroid	Betamethasone (injectable), dexamethasone (injectable), prednisone (oral)	10.0 (24/240)
4	Proton pump inhibitor	Omeprazole, pantoprazole	9.6 (23/240)
5	Benzodiazepines	Alprazolam, clonazepam, diazepam	8.3 (20/240)
6	Muscle relaxant	Cyclobenzaprine, carisoprodol	5.8 (14/240)
7	First-generation antihistamine	Dexchlorpheniramine, promethazine	5.4 (13/240)
8	Antispasmodic	Scopolamine	3.8 (9/240)
9	Antihypertensive	Methyldopa, clonidine, nifedipine	3.8 (9/240)
10	Tricyclic antidepressants	Amitriptyline, clomipramine, imipramine	3.8 (9/240)

According to the univariate analysis in **Table 4**, the patient factors associated with PIM prescription were female sex (PR: 1.56; 95% confidence interval, CI: 1.20-2.03); being single, widowed or divorced (PR: 1.31; 95% CI: 1.06-1.62); reporting insomnia (PR: 1.36; 95% CI: 1.10-1.68); having an allotted consultation length < 10 minutes (PR: 0.72; 95% CI: 0.59-0.90); and receiving a prescription of polypharmacy (PR: 1.68; 95% CI: 1.37-2.05). However, after adjusting for potential confounders in the multivariate analysis, only the association with receiving polypharmacy remained statistically significant (PR: 1.50; 95% CI: 1.06-2.12).

**Table 4:** Patient-related factors associated with prescription of potentially inappropriate medications

Characteristics		PIM		Univariate analysis		Multivariate analysis*	
		Yes % (n/N)	No % (n/N)	PR (95% CI)	P-value	Adjusted PR (95% CI)	P-value
Sex							
	Male	33.1 (45/136)	66.9 (91/136)	1.0 1		1	
	Female	51.6 (145/281)	48.4 (136/281)	1.56 (1.20-2.03)	< 0.001	1.32 (0.90-1.94)	0.154
Age in years, median (IQR)		68.0 (64.0-74.25)	69.0 (65.0-75.0)		0.337**		
Schooling							
	Literate	47.9 (113/236)	52.1 (123/236)	1		1	
	Illiterate	43.0 (77/179)	57.0 (102/179)	1.11 (0.90-1.38)	0.325		
Marital status							
	Married	39.6 (86/217)	60.4 (131/217)	1		1	
	Single, widowed or divorced	52.0 (104/200)	48.0 (96/200)	1.31 (1.06-1.62)	0.011	1.12 (0.82-1.54)	0.463
Ethnicity							
	White	39.2 (40/102)	60.8 (62/102)	1			
	Mixed	48.5 (149/307)	51.5 (158/245)	1.24 (0.95-1.62)	0.102		
Personal income							
	1 minimum salary	43.3 (68/157)	56.7 (89/157)	1			
	< 1 minimum salary	46.9 (122/260)	53.1 (139/260)	1.07 (0.58-1.97)	0.473		
Health plan							
	Yes	42.9 (6/14)	57.1 (8/14)	1			
	No	45.7 (184/403)	54.3 (219/403)	1.07 (0.58-1.97)	0.836		
Cognitive impairment							
	No	44.0 (91/207)	56.0 (116//207)	1			
	Yes	47.4 (99/209)	52.6 (110/209)	1.08 (0.87-1.33)	0.485		
ADL impairment							
	No	43.1 (116/269)	56.9 (153/269)	1			
	Yes	50.0 (74/148)	50.0 (74/148)	1.16 (0.94-1.43)	0.224		
IADL impairment							
	No	45.5 (15/33)	54.5 (18/33)	1			
	Yes	38.0 (19/50)	62.0(31/50)	0.84 (0.50-1.40)	0.499		
Visual impairment							
	No	44.8 (77/172)	55.2 (95/172)	1			
	Yes	46.1 (113/245)	53.9 (132/245)	1.03 (0.83-1.28)	0.848		
Hearing impairment							
	No	45.8 (125/273)	54.2 (148/273)	1			
	Yes	45.1 (65/144)	54.9 (79/144)	0.99 (0.79-1.23)	0.899		
Insomnia							
	No	38.8 (83/214)	61.2 (131/214)	1			
	Yes	52.7 (106/201)	47.3 (95/201)	1.36 (1.10-1.68)	0.004	1.29 (0.95-1.75)	0.105
Chronic pain							
	No	41.0 (55/134)	59.0 (79/134)	1			
	Yes	47.7 (135/283)	52,3 (148/283)	1.16 (0.92-1.47)	0.227		
Fall history							
	No	41.4 (70/169)	58.6 (99/169)	1			
	Yes	49.2 (118/240)	50.8 (122/240)	1.19 (0.95-1.48)	0.122		
Hospitalization							
	No	45.4 (165/366)	54.6 (200/366)	1			
	Yes	47.1 (24/51)	52.9 (27/51)	1.04 (0.76-1.42)	0.790		
Multimorbidity							
	No	59.3 (16/27)	40.7 (11/27)	1			
	Yes	44.6 (174/390)	55.4 (216/390)	1.16 (0.93-1.46)	0.178		
Polypharmacy							
	No	40.9 (142/347)	59.1 (205/347)	1		1	
	Yes	68.6 (48/70)	31.4 (22/70)	1.68 (1.37-2.05)	< 0.001	1.50 (1.06-2.12)	0.022
Companion attending consultations							
	Yes	41.0 (34/83)	59.0 (49/83)	1			
	No	46.5 (155/333)	53.5 (178/333)	1.14 (0.86-1.51)	0.361		
Length of consultation ≤ 10 minutes							
	No	53.3(105/197)	46.7 (92/197)	1			
	Yes	38.6(85/220)	61.4 (135/220)	0.72 (0.59-0.90)	0.003	0.77 (0.57-1.05)	0.095

* Dependent variable was PIM; model: (intercept), sex, marital status, insomnia, multimorbidity, polypharmacy and consultation length < 10 minutes

** Mann-Whitney U test. One minimum monthly wage was equivalent to US$ 283.00.

ADL = activity of daily living; CI = confidence interval; IADL = instrumental activity of daily living; PIM = potentially inappropriate medication; PR = prevalence ratio; IQR = interquartile range.

Regarding physician-related factors (**Table 5**), the univariate analysis showed that prescription of PIMs showed positive associations with the number of patients attended (*P* < 0.001) and the number of prescriptions per physician (P < 0.001). Furthermore, younger physicians (length of time in medical practice < 10 years) prescribed more PIMs than older physicians (PR: 1.22; 95% CI: 0.99-1.51).

**Table 5:** Physician-related factors associated with prescription of potentially inappropriate medications

Characteristics		Prescription of PIMs		Univariate analysis	
		Yes % (n/N)	No % (n/N)	PR (95% CI)	P-value
Number of physicians		86.8 (46/53)	13.2 (7/53)		
Sex					
	Male	84.6 (22/26)	15.4 (4/6)	1	
	Female	92.0 (23/25)	8.9 (2/6)	1.09 (0.89-1.33)	0.413
Length of time in medical practice < 10 years					
	No	78.6 (22/28)	21.4 (6/28)	1	
	Yes	96.0(24/25)	4.0(1/25)	1.22 (0.99-1.51)	0.060
Specialty					
	Yes	91.7 (11/12)	8.3 (1/12)	1	
	No	89.2 (33/37)	10.8 (4/37)	1.03 (0.84-1.26)	0.805
Type of primary care unit					
	FHU	88.2 (30/34)	11.8 (4/34)	1	
	PHU	84.2 (16/19)	15.8 (3/19)	1.05 (0.83-1.32)	0.678
Number of patients attended per physician, median (IQR)		11.5 (7.25-15.0)	1.0 (1.0-1.0)	–	< 0.001*
Number of prescriptions per physician, median (IQR)		10.0 (6.25-12.0)	1.0 ± 0 (1.0-1.0)	–	< 0.001*

* Mann-Whitney U test.

PHU = primary healthcare unit; CI = confidence interval; FHU = family health unit; PIM = potentially inappropriate medication; PR = prevalence ratio; IQR = interquartile range.

## DISCUSSION

In this study, a total of 45.3% of the patients received a prescription of ≥ 1 PIM, as determined using the Brazilian criteria.^4^ In addition, 86.8% out of 53 physicians prescribed ≥ 1 PIM. The strongest patient-related factor was polypharmacy, while the number of patients attended, number of prescriptions per physician and length of time in medical practice < 10 years were significant physician-related factors. To the best of our knowledge, this study is so far one of the largest studies to examine PIM prescriptions among older adults in Brazil, and the first study to evaluate physician-related factors associated with PIM prescriptions in Brazil.

A systematic review of 19 studies conducted in different countries reported that one in five medications prescribed to older people within primary care was inappropriate.^23^ The prevalence of PIM prescription within primary care varied between countries and according to the criteria used: totals of 41% in the United States (Beers 2003 criteria);^5^ 36% in Ireland (Screening Tool for Older Persons’ potentially inappropriate Prescriptions [STOPP] criteria, 2008 version);^14^ 34.8% in Norway (Norwegian General Practice criteria, [NORGEP]);^33^ 27.3% in Serbia (STOPP criteria, 2008 version);^34^ 37% in Canada (Beers 2012 criteria);^15^ 34.7% in the Netherlands (Dutch version of the STOPP criteria);^35^ 26.4%, 37.4% and 13.7% in Germany, defined by the 2015 Beers criteria, the European Union (EU) (7)-PIM list [EU(7)-PIM] and the PRISCUS list, respectively;^10^ and 48.5% in Spain (STOPP criteria, version 2015).^36^ These data show that, in several countries, the criteria specified and local prescription habits influenced the prevalence of PIM prescription.

Like in several other studies, it was found in the present study that the pharmacological classes of PIM that were most prescribed were benzodiazepines,^2^^,^^5^^,^^10^^,^^14^^,^^15^^,^^18^^,^^33^^,^^34^^,^^37^ nonsteroidal anti-inflammatory drugs (NSAIDs),^2^^,^^5^^,^^11^^,^^14^^,^^17^^,^^18^^,^^21^^,^^34^ proton pump inhibitors,^11^^,^^14^^,^^18^ first-generation antihistamines,^2^^,^^5^^,^^11^^,^^14^^,^^18^^,^^21^^,^^33^ muscle relaxants,^2^^,^^5^^,^^15^^,^^18^^,^^33^ tricyclic antidepressants,^2^^,^^5^^,^^11^^,^^14^^,^^15^^,^^33^ antihypertensives,^2^^,^^14^^,^^18^^,^^33^ and oral antihyperglycemics.^5^^,^^14^^,^^15^^,^^18^^,^^33^^,^^34^ However, in contrast to previous research, we observed large numbers of prescriptions of systemic corticosteroids, particularly injectables. In our study, approximately 70% of the older patients suffered from chronic pain. They probably received a prescription of systemic corticosteroids to manage chronic pain, even though this prescriptive approach for older adults is not supported by any data in the literature.^38^^–^^40^ Similarly, although systemic corticosteroids are not recommended for low back pain management,^41^^,^^42^ a survey reported that nearly 25% out of 720 American physicians opted for systemic corticosteroids as their initial approach for managing acute low back pain-related sciatica.^43^

As reported in the literature, the most important predictor of inappropriate prescribing to older individuals is prescription of polypharmacy.^14^^,^^17^^,^^32^ Depending on the circumstances, including why and how medications are being administered, polypharmacy may be either appropriate (the potential benefits outweigh the potential harm) or inappropriate (the potential harm outweighs the potential benefits). Therefore, the main anti-PIM intervention might consist of avoidance of inappropriate polypharmacy through applying the principles of rational and appropriate prescription and deprescribing medications that are no longer needed.^44^^,^^45^

With regard to the physicians in the present study, almost all of them (90%) prescribed ≥ 1 PIM. In addition, physicians who attended to more patients and wrote more prescriptions were more likely to prescribe ≥ 1 PIM. Furthermore, PIM prescription was 22% more prevalent among younger physicians. According to a German study, the reasons for PIM prescription by family physicians included the following: their limited knowledge of PIMs; limited applicability of detecting PIM lists (like the Beers and STOPP criteria) in daily practice; shortages of time; lack of alternative medications; and bad experiences relating to medication changes.^46^

The findings from this cross-sectional study warrant future studies to assess interventions that can help improve the appropriateness of drug prescriptions to elderly people. The topics that need to be addressed include education programs targeted to family physicians and the use of decision support systems in primary care settings.

### Limitations

The findings of the present study might be explained by the primary care doctors’ unfamiliarity with PIMs and the limited availability of safer therapeutic alternatives for older people through SUS.^47^ However, our study was not designed primarily to investigate factors relating to the prescribing physician and, therefore, our sample was not calculated to address these factors. Moreover, the variables relating to physicians were limited to data held by the Federal Council of Medicine, which therefore left some gaps that future investigations should address.

## CONCLUSION

Half of the patients in our current study received ≥ 1 PIM prescription and nine in every ten primary care physicians prescribed ≥ 1 PIM. Regarding the patient-related factors, polypharmacy was the strongest factor associated with higher prevalence of PIM prescriptions. Among the physician-related factors, we found that the number of patients attended per physician, the number of PIM prescriptions per physician and length of time in medical practice < 10 years were positively associated with prescription of PIMs. Moreover, NSAIDs, oral hypoglycemic agents and injectable systemic corticosteroids were more commonly prescribed than the other pharmacological classes of PIMs. Future interventions targeting all physicians, especially the younger ones, are required in order to promote prescription of appropriate medications to older patients.
